# 3D modelling of γδ T‐cell immunotherapy

**DOI:** 10.1002/ctm2.877

**Published:** 2022-05-11

**Authors:** Caroline M. Hull, John Maher

**Affiliations:** ^1^ Leucid Bio Ltd. Guy's Hospital Great Maze Pond London UK; ^2^ CAR Mechanics Lab Guy's Cancer Centre School of Cancer and Pharmaceutical Sciences King's College London Great Maze Pond London UK; ^3^ Department of Immunology Eastbourne Hospital Eastbourne East Sussex UK

1

Adoptive T‐cell therapy has shown promise in recent years, particularly in blood cancers using autologous CAR αβ T cells that target CD19.^1^ However, γδ T‐cell therapies have many advantages compared to their αβ T‐cell counterparts. Tumour infiltration by γδ T cells is one of the best predictors of improved prognosis in numerous solid tumours.^2^, ^3^ In addition, γδ T cells are equipped with an array of innate‐like receptors that recognize several phosphoantigens and stress ligands, theoretically reducing the chance of immune evasion by tumour cells.^4^ When expanded in the presence of the prototypic tumour‐associated immunosuppressive cytokine, transforming growth factor‐ß, γδ T cell intrinsic anti‐tumour activity is further enhanced, accompanied by resistance to suppressive effects of this cytokine.^5^ Furthermore, the lack of HLA restriction of γδ T cells negates their ability to induce graft versus host disease, when used in the allogeneic setting.^6^

The γδ T‐cell subset that has been most frequently studied for use as a cell therapy is blood derived, the majority of which express a Vγ9Vδ2 T‐cell receptor. However, the anti‐tumour activity of these cells when tested in 2D culture models has not been replicated in clinical trials.^7^ Cancer cells cohabit and interact with mesenchymal stroma and leukocytes, collectively creating a highly immunosuppressive tumour microenvironment (TME). Immunosuppressive cells found in this ecosystem include cancer‐associated fibroblasts (CAFs), regulatory T cells and a range of suppressive myeloid cell types. This complexity is not recapitulated in simple 2D cancer model systems. While some immunocompetent mouse models may mimic attributes of the human TME, Vγ9Vδ2 T cells are not found in rodents. Consequently, there is a need for more relevant tumour model systems with which to test efficacy and identify obstacles to successful clinical implementation of γδ T‐cell immunotherapy.

In this issue, Ou et al. have developed four distinct uni‐, bi‐ and multicellular melanoma models to study infiltration, activation and tumour cell killing by γδ T cells. Using this approach, they also highlight a number of inhibitory pathways deployed by the TME and point towards pharmacological solutions that may enhance clinical success of γδ T‐cell immunotherapy. Ou et al. first demonstrated that freshly isolated peripheral blood derived γδ T cells can infiltrate tumour spheroids faster and in greater numbers than αβ T cells. Although the mechanisms that underpin this finding are unclear, this establishes γδ T cells as a type of ‘rapid response unit’ to cancer. However, infiltrated γδ T cells promptly upregulate a range of inhibitory checkpoint proteins (CTLA‐4, PD‐1, PD‐L1) accompanied by lowered CD69 and NKG2D expression, in keeping with an exhausted phenotype. Inclusion of CAFs in the tumour spheroids to yield a bicellular model increases this inhibitory response, while further compromising γδ T‐cell recruitment. However, these suppressive effects of the TME could be overcome by addition of checkpoint blockade, using anti‐CTLA‐4 and anti‐PD‐1 monoclonal antibodies. As a result, infiltration by γδ T cells, IFN‐γ production and tumour cytolytic activity were all increased. Comparing γδ T cells outside the spheroids to those inside suggests that infiltrating γδ T cells either adopt an effector memory phenotype during migration, or that effector memory γδ T cells preferentially infiltrate the spheroids. Additional investigation is warranted to uncover mechanisms by which CAFs exert these inhibitory effects and how the change in memory phenotype impacts γδ T‐cell function.

Importantly, Ou et al. also found that ex vivo expanded γδ T cells could infiltrate multicellular spheroids in which tumour, CAFs and other mesenchymal cell types were present. Once again, this was accompanied by an increase in inhibitory checkpoint protein expression. To further mirror the TME of solid tumours, Ou et al. next established melanoma patient‐derived organoids (MPDOs), which differ to multicellular spheroids in that they also contain immune cells such as tumour infiltrating lymphocytes (TILs) (Figure 1). In all four 3D models, including the MPDOs, addition of anti‐CTLA‐4 and anti‐PD‐1 monoclonal antibodies significantly enhanced the infiltration, IFN‐γ release and tumour cytolytic activity of in vitro expanded γδ T cells. Further interrogation of how γδ T‐cell infiltration along with checkpoint blockade influences other immune cells, including NK cells and TILs within the MPDO, would enhance our understanding of the mechanisms by which this combinatorial approach relieves suppressive actions of the TME.

**FIGURE 1:**
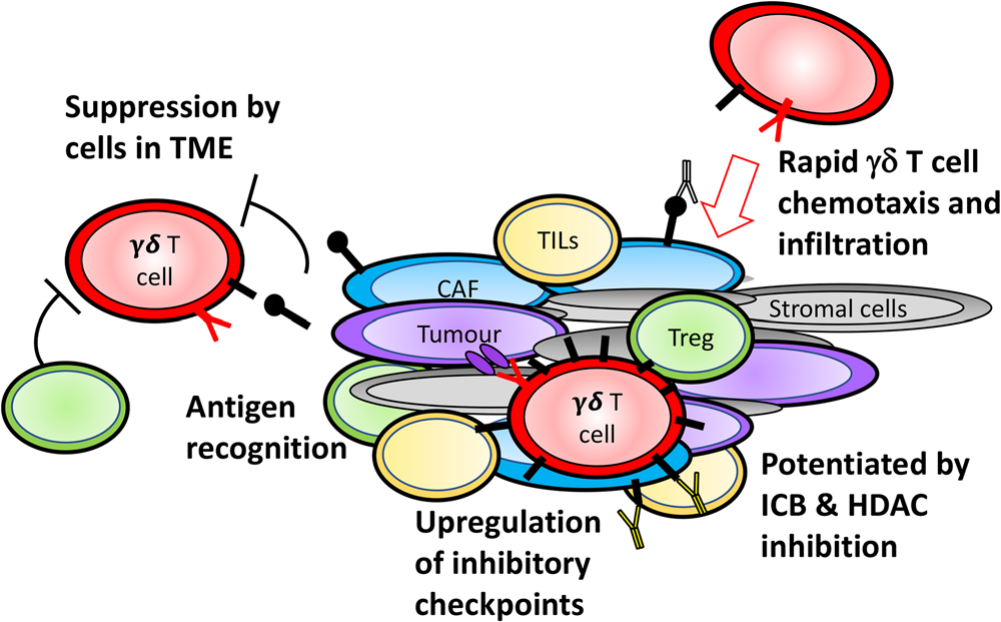
Melanoma patient‐derived organoids (MDPOs) allow complex modelling of γδ T‐cell function in vitro. Function of adoptively transferred human γδ T cells cannot be adequately characterized in 2D in vitro tumour co‐cultures or in immunocompetent mouse models. To better model the complexity of the TME, MPDOs represent a more authentic model that allow testing of the interaction between γδ T cells and tumour cells in their natural environment. Such models have revealed the rapid nature of γδ T‐cell influx into tumours, followed by upregulated expression of immune checkpoint molecules. Therapeutic role of immune checkpoint blockade (ICB) and histone deacetylase (HDAC) inhibition have also been revealed using these elegant systems

Epigenetic factors also play a key role in maintaining the immunosuppressive TME.^8^ In addition to checkpoint blockade with monoclonal antibodies, Ou et al. undertook a small molecule screen and found that histone deacetylase inhibitors, notably entinostat and vorinostat, also countered inhibitory TME effects on γδ T cells. These agents increased expression of the NKG2D ligands, MICA and MICB, in melanoma‐fibroblast bicellular spheroids. In addition, PDL1/2 expression on CAFs and tumour cells was decreased, while γδ T‐cell cytotoxicity was enhanced. Epigenetic modifiers also increased expression of NKG2D, CXCR3 and CD107a on γδ T cells while decreasing expression of checkpoint molecules including PD‐1. These data highlight the importance of chemokine receptor expression by γδ T cells and implicate CXCR3 in efficient γδ T‐cell recruitment in vivo. Analysis of the impact of small molecules on patient‐derived γδ T cells already within the MPDO would also be of interest.

Collectively, these findings confirm that upon recruitment to the TME, γδ T cells adopt an exhausted phenotype that can be overcome by addition of checkpoint inhibitors and epigenetic modifiers. The influence that small molecules have on adoptively transferred γδ T cells is an interesting point to explore as the utility of entinostat and vorinostat in melanoma is already being investigated in the clinic.^9^, ^10^ The effect of epigenetic modifiers on stress ligand expression (e.g. MICA/B) by healthy tissue also warrants further consideration. Increased NKG2D ligand expression on healthy tissues could potentially unmask toxicity when combined with high doses of γδ T cells due to on‐target off‐tumour cytotoxicity. Consequently, modelling of the safety profile of such combination therapies also requires further investigation.

## CONFLICT OF INTEREST

J.M. is CSO, scientific founder and shareholder of Leucid Bio, is a member of the scientific advisory board of Arovella Therapeutics Ltd and has undertaken consultancy work for Bristol‐Meyers‐Squibb, Juno, Celgene, Ellipses Pharma and Biotest. C.H. is an employee of Leucid Bio.
